# Changes in admission to long-term care institutions in the Netherlands: comparing two cohorts over the period 1996–1999 and 2006–2009

**DOI:** 10.1007/s10433-016-0393-0

**Published:** 2016-08-08

**Authors:** Peter Alders, Hannie C. Comijs, Dorly J. H. Deeg

**Affiliations:** 1 Institute of Health Policy and Management, Erasmus University Rotterdam, PO Box 1738, 3000 DR Rotterdam, The Netherlands; 2 Department of Psychiatry, EMGO Institute for Health and Care Research, VU University Medical Center and GGZ inGeest, Amsterdam, The Netherlands; 3 Department of Epidemiology and Biostatistics, EMGO Institute for Health and Care Research, VU University Medical Center, Amsterdam, The Netherlands

**Keywords:** Institutional care, Older adults, 10-year change, Blinder–Oaxaca decomposition

## Abstract

Using data from two cohorts, we examine to what extent a decline in institutional care in the Netherlands is associated with changes in the need for care and/or societal factors. We compared older adults, aged 65–89, who were admitted to a long-term care (LTC) institution in the period 1996–1999 and 2006–2009. Using the Andersen model, we tested per block of predisposing, enabling and need factors, which factors were significant predictors of admission to institutional care. With a Blinder–Oaxaca decomposition regression, we decomposed the difference in admission to an LTC institution between the period 1996–1999 and 2006–2009 into a part that is due to differences in health needs and other factors such as effect of policy, social values, and technology. Between 1996 and 2006, the percentage of co-residing partners and income increased and the average level of loneliness decreased significantly. The prevalence of disability, chronic diseases, however, increased. Whereas the care by partners declined, the formal care by professionals increased. Although the observed decline in the admission rate to institutional care was relatively small across the 10 years (from 5.3 % in 1996–1999 to 4.5 % in 2006–2009, a 15 % decrease), the probability of admission in 2006–2009 was relatively much lower when accounting for changes in the health and social conditions of the participants: the probability was 1.7–2.1 % point lower for adults in the period 2006–2009 compared to 1996–1999, a 32–40 % decrease. Our results show that the decline in the admission rate to LTC institutions is not the result of changes in need. The decline is suggested to be the combined effect of changes in policy, technological advances and changes in social norms.

## Introduction

Older European adults have strong preferences not to be served in institutional care (Eurobarometer Surveys 2007). Moreover, the Organisation for Economic Co-operation and Development (OECD) expects upward pressure on the demand for long-term care (LTC) services and, as a consequence, the human and financial resources necessary to provide LTC services. Over the past decades, nearly all OECD countries have been encouraging “ageing in place” policies (Francesca et al. 2011). However, we know little about the way public policy affects use of institutional care, whether changes in admission to institutional care is driven by changes in the health situation and support system or societal factors. Considerable differences were found between eight European countries in characteristics of people with dementia who had been recently admitted to institutional dementia care (Verbeek et al. 2015). Many studies investigate predictors of institutionalization (Gaugler et al. 2007, 2009; Luppa et al. 2010). Most often factors that cannot be affected by government policy, such as activities of daily living (ADL) problems, cognitive impairments, and lack of a social network, are found as predictors.

The Netherlands might be an interesting country to study the effect of government policy and cultural change on the rate of admission to institutional care. Although need factors would predict a higher rate of institutional use in Germany, in 2004 the percentage of people over 65 in institutions in the Netherlands was almost double the percentage in Germany (Alders et al. 2015). Furthermore, the percentage of people over the age of 65 years, living in an institution dropped from 7.2 % in 2004 to 6.5 % in 2010 in the Netherlands. This trend is similar to the trend in other OECD countries with—relatively—high levels of institutional care: in the period 2000–2010 the percentage of persons over 65 in LTC institutions declined in Sweden from 7.7 to 5.3, in Norway from 6.0 to 5.5, in Switzerland from 6.8 to 6.0 and in Denmark from 5.4 in 2006 to 4.5 in 2010. However, in OECD countries with lower levels of institutional care, the level of institutional care was stable or (slightly) increased; in Germany the percentage increased from 3.7 in 2000 to 3.8 in 2010, in Canada the level was stable at 3.4 and in France it increased from 3.5 (in 2003) to 4.3 in 2010 (OECD 2014). The decline in Denmark coincided with a decline in severe disability, whereas Sweden reports an increase in severe disability (Lafortune and Balestat 2007). In this study, we pursue to provide insights in these trends by investigating the Dutch situation over a longer period of time.

### Background

Admission to an LTC institution is generally related to concerns about safety for a person (for instance a fall) or for his or her environment (for instance a risk of causing a fire accident or a frail spouse) and the inability to guarantee personal hygiene. Such limitations mirror the functions that LTC institution provide and that are difficult to fulfil at home: 24 h unplanned care, continuous supervision to ensure a safe, clean and organized place, specialized care concerning ADL, instrumental ADL or chronic diseases, and company of other people.

According to the Andersen healthcare utilization model (1995), the use of health services is determined by three dynamics: predisposing factors (such as age and education), enabling factors (such as family support and income) and need (such as poor physical or mental health and activity limitations). Predisposing factors relate to older people’s attitudes and willingness to ask for care, regardless of their need for care. Enabling factors are factors that stimulate or hamper the utilization of health care. Need variables are primarily related to the physical and mental condition of older adults. Additionally, the health care system was explicitly included in this model by Aday and Andersen (1974), giving recognition to the importance of national health policy and the resources and their organization in the health care system as important determinants of the population’s use of services.

Regarding predisposing factors, persons will differ in their likelihood of admission to an LTC institution, depending on their capacity to organize care, and their social norms and preferences by whom they want to be cared for. Subsumed under enabling factors, partners and family members play a pivotal role in the care system of their spouse or family member. Their commitment and time allocated to informal care can make the difference in providing hygiene, safety and a valuable social life. Other enabling factors, such as income and wealth, might make it easier to organize extra private care and support or live in a house where specialized care can be delivered more easily. The most powerful predictors for admission to an LTC institution are need factors (Gaugler et al. 2007). Informal caregivers in eight European countries state mainly patient-related reasons for institutionalization, such as neuropsychiatric symptoms, care dependency and cognition. Besides patient-related reasons, caregiver burden and the inability of the informal caregiver to care for the patient were stated as reasons (Afram et al. 2014).

Over the last decades, several trends might have affected the need for care and the admission rate to institutional care. Life expectancy at age 65 increased from 15.1 to 16.8 years for men and 19.5 to 20.4 for women over the period 1996–2006 (Statistics Netherlands 2015). This means for couples, they have a higher probability to have a partner around when one needs help. Lakdawalla and Philipson (2002) find evidence that growth in elderly males causes couples to stay married longer and raise the supply of spousal care: a ten percentage point increase in the ratio of men per woman appears to reduce the per capita stock of nursing home residents by as much as 16 %. Between 1992 and 2012, formal home care use increased slightly while there was a large decrease in the use of informal care in the Netherlands (Swinkels et al. 2015). In addition, in general younger cohorts are better educated and have a relatively higher income, which makes it easier to obtain paid support in the household. Life-style behaviour changed, smoking declined, but relatively more people became obese. In the Netherlands, in the period 1990–2008 prevalence rates of chronic diseases increased in community-living older people, whereas prevalence rates of activity limitations were stable or slightly decreased depending on the definition (Hoeymans et al. 2012). Other research showed an increase in the prevalence of mild activity limitations, but not in severe activity limitations in the Dutch older population over the period 1992–2009 (Galenkamp et al. 2013).

Government policy and social norms have been suggested as important explanatory factors of the relatively high level of institutional care in the Netherlands, based on a comparison of the cases of the Netherlands and Germany (Alders et al. 2015). In 1995, admission to an LTC institution became less expensive for people with assets as the government ceased means testing (Alders et al. 2015). Personal budgets were introduced in 1995. Since 1999, after a court ruling that older adults can exercise a right for care when eligible, the level of home care increased and almost doubled in the following decade (Schut and Van den Berg 2010). Improvements in care and technology gave people more possibilities to age-in-place. Technology, home automation, telehealth services, and ‘ambient intelligence’ are increasingly becoming tools to support and monitor older adults with or without cognitive impairments, by improving their sense of safety and security as a means to support ageing-in-place (van Hoof et al. 2011). The number of joint replacement surgeries and the use of nonsteroidal anti-inflammatory drugs to treat arthritis and antihypertension medication increased (Cutler 2001).

Note that a decline in the admission rate to LTC institutions caused by need factors has an opposite effect on the health situation of the people living in the community from the situation that the decline is caused by factors as technological change or a change in social norms. A drop in the admission to LTC institutions caused by a change in social norms, results in more frail people living in the community, whereas a decline in need factors implies a relatively more healthy population.

To obtain a better understanding of the trend in admission to LTC institutions, we used data from the Longitudinal Aging Study Amsterdam (LASA) to compare people who were admitted to an institution in the period 1996–1999 with those admitted in 2006–2009. We examine whether a decline in LTC institution use is associated with changes in enabling and predisposing factors, such as an improved educational level or better income, changes in the need for care, or that a decline instead might be attributed to factors such as technological advances in housing, government policy, or social norms.

## Methods

### Sample

LASA is an ongoing study on predictors and consequences of changes in physical, cognitive, emotional, and social functioning of older people. The original LASA cohort is based on a nationally representative sample of adults aged 55–85 years in 1992–1993 (years of birth 1908–1937, *N* = 3107), recruited in three geographic regions in the Netherlands. These regions were selected to achieve an optimal representation of the older Dutch population. Follow-up cycles were carried out every 3–4 years. An additional cohort was recruited from the same sampling frame in 2002/2003 (year of birth 1938–1947, *N* = 1002).

Trained interviewers who visit respondents at their home perform the measurements. Participants who were not able or refused to participate in the complete face-to-face interview were asked to participate in a 15-min telephone interview. For participants who were not able to do a telephone interview, a proxy respondent was asked to answer a set of questions. The sampling and data collection procedures have been described in more detail elsewhere (Huisman et al. 2011). Attrition, respectively in the period 1996–1999 and 2006–2009, was primarily caused by mortality. In 1999, 13.5 % had died in the previous 3 years and 5.0 % had dropped out for other reasons. In 2009, 11.2 % had died in the previous 4 years and 4.8 % had dropped out for other reasons.

At the baseline interview, respondents were asked for their informed consent. Also, consent forms were signed in which people give permission to LASA to gather additional medical information. The Medical Ethical Board of the VU University Medical Center approved the study design.

**Fig. 1:**
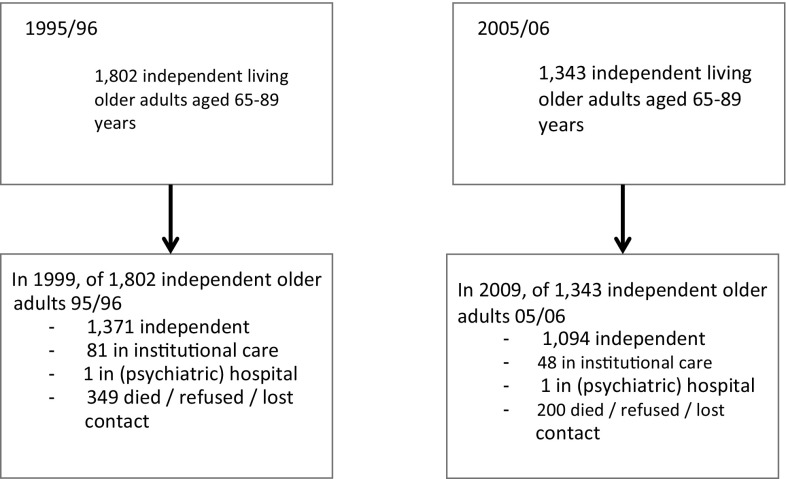
*Flowchart* respondents 65–89 years old in cycles 1995/1996–1999 and 2005/2006–2009

We compared the admission rate to LTC institutions from two cycles 10 years apart: cycle 1995–1996 and cycle 2005–2006. We restricted the study samples to people living in the community and observed who were living in an LTC institution 3 years later, in 1999 and 2009, respectively. To compare the same age groups, we restricted the age range of our study to 65–89. The sample sizes were 1452 for the 1995/1996 cohort and 1142 for the 2005/2006 cohort. From the 1995/1996 cohort, 81 persons were in institutional care in 1999; from the 2005/2006 cohort 48 persons were in institutional care in 2009 (Fig. 1).

### Measures

The dependent variable is the admission to an LTC institution in the period 1996–1999 or 2006–2009. In the Netherlands, citizens can be admitted to institutional care when they need permanent supervision or need a sheltered residence (Centre for Care Assessment 2013).

As potential explanatory variables we used the predisposing variables age, sex and education. Education was categorized into three levels: low (elementary school not completed, elementary school, lower vocational education), intermediate (general intermediate, intermediate vocational, general secondary education) and high level of education (higher vocational, college or university education). Enabling factors used were the level of income, living with a partner (yes/no) and having children (yes/no). For income, the average income of the respondent’s neighbourhood was used. Income was measured in five categories (1–5: minimum, minimum–modal, modal, modal–twice modal, >twice modal). We considered formal and informal care as enabling factors as well. Formal care can be household help as well as personal care. Formal care is delivered by professionals who do not have a social relationship with the older person but who deliver care as part of their paid work. We distinguished two forms of informal care. Informal care provided by the partner and provided by persons with whom another social relationship exists, i.e. child, other relative, neighbour or other non-kin. Two questions were asked on the use of household and personal care: ‘Do you receive help with household tasks (e.g. shopping, gardening, cooking, cleaning, taking garbage out and filling out forms) and personal care (e.g. washing, bathing or showering, dressing, going to the toilet, getting up and sitting down), and if so, from whom?’ Respondents could report different types of informal and formal care helpers.

The need variables that we used were self-reported chronic diseases, ADL disability, cognitive functioning, depressive symptoms and loneliness. Self-reported chronic diseases included: chronic lung disease (COPD), heart disease, peripheral artery disease, diabetes mellitus, stroke, cancer, incontinence, rheumatoid and osteoarthritis or any other chronic disease, defined as a disease of which symptoms and/or treatment had been present for at least 3 months (Kriegsman et al. 1996). ADL disability was assessed by asking whether respondents had difficulty performing six activities: getting up from a chair, dressing, walking down and up a staircase of 15 steps without resting, using one’s own or public transportation, walking outside for 5 min without stopping, and cutting one’s toenails (Mc Whinnie 1980). Furthermore, as a need factor we use whether the respondent was hospitalized over the last 6 months.

For the cognitive state, we used a variable indicating probable dementia (yes/no), based on a significant decline in cognitive functioning over a period of 6 years as measured with the mini–mental state examination (MMSE; Folstein et al. 1975; Tombaugh and McIntyre 1992) or a shortened telephone informant questionnaire on cognitive decline in the elderly (IQCODE; Jorm and Korten 1988), data from general practitioners (GPs), and the interviewers (Van den Kommer et al. 2008). The MMSE is widely used as a tool for monitoring change in global cognitive functioning. This version included the following items: year, day of the week, month, two streets in the neighbourhood, address, repeating three words, the highest score on either subtracting (100−7) or spelling backwards, remembering three words. For participants for whom only proxy data were obtained, an abbreviated version of the IQCODE was administered. This version has been recommended for use as an efficient rating scale for clinical assessment of dementia (de Jonghe 1997). Six items in which decline over the past 10 years was enquired are included in the short version of the IQCODE: remembering conversations a few days later, remembering his or her address and telephone number, knowing how to work familiar machines around the house, making decisions on everyday matters, handling money for shopping, handling financial matters. The items are scored on a five-point scale: 1 = much better, 3 = no change, 5 = much worse. Sum scores range from 6 to 30. Persistent cognitive decline was determined by comparing the score on the (abbreviated) MMSE in the baseline years (1996 and 2006) with the MMSE score at the previous measurement cycles (1993 and 2002, respectively), and defined as more than two standard deviations below the average decline of the total sample (Altman 1999). Persistent cognitive decline on the IQCODE was defined by a minimum score of 28 (i.e. the maximum score of 5 on at least four areas, and a score of 4 on the remaining two areas). Finally, if no longitudinal measurements of the (abbreviated) MMSE or the IQCODE were available, cognitive decline was considered present when the interviewer recorded ‘dementia’ as the reason for loss-to-follow-up. In addition, information from GPs concerning dementia diagnosis by GP or specialist were used. As measure of depressive symptoms, we used a short version from the Center for Epidemiological Studies Depression scale, i.e. the items “was bothered”, “felt depressed”, “felt fearful” and “felt lonely”, which enabled to use both the results of the telephone interview and the face-to-face interviews (Radloff 1977). The loneliness measure contains 11 statements about loneliness, with a scale from 0 (=no loneliness) to 11 (=severe loneliness) (de Jong Gierveld and Kamphuis 1985).

### Statistical analyses

The samples of 1996 and 2006 were pooled. For descriptive analyses, weights were applied to bring the age–sex distribution of the 1996 sample in accordance with the 2006 sample. With the dummy variable “year” the baseline year was measured: year = 0 in 1996 and year = 1 in 2006. This dummy picks up the effect of policy, societal changes and technological changes, although we cannot disentangle the effects of these variables separately with the data and analyses used in this study. We used logistic regression to find the predictors of institutional care. Using the Andersen model, we tested per block predisposing, enabling and need factors, to determine which factors were significant predictors of admission to institutional care. A covariate was selected for inclusion in multivariable analyses when it was associated with admission to an LTC institution (*p* < 0.10). We performed a sensitivity analysis, by relaxing the inclusion criterion to *p* < 0.20. Moreover, in the final model we removed variables with a significance level of *p* > 0.50 to obtain a more parsimonious model.

Furthermore, with the remaining variables, we carried out a Blinder–Oaxaca decomposition regression according to the method of Yun (2004). With this analysis, we decompose the difference in admission to an LTC institution between the period 1996–1999 and 2006–2009 into a part that is due to differences in the magnitudes of the determinants on the one hand (hereafter “due to endowments”), and differences in the effects of these determinants (hereafter “due to effects”), on the other hand. For example, in the period 2006–2009, the disability level of the older adults living independently might be different from the disability level of older adults in 1996–1999, but as well the probability that a disability results in an admission to an LTC institution might have changed.

## Results

The descriptive characteristics were weighed for age and gender. For both baseline years, the average age was 74.1 years old and 56.8 % was female. The 2006 sample showed a significantly higher percentage of co-residing partners, neighbourhood income level and a lower average level of loneliness than the 1996 sample (Table 1). However, greater prevalences of disability and chronic diseases were observed in the sample of 2006 than in the 1996 sample. Significantly more people reported heart diseases, diabetes, osteoarthritis, cancer and incontinence. The percentage of people with at least two chronic diseases and conditions increased. Whereas significantly less people received informal care by the partner, significantly more people received formal care. The percentage of older adults with dementia showed no significant change.

**Table 1:** Descriptive characteristics of participants (age between 65 and 89) in baseline cycles 1996 and 2006

	Total *N*	Baseline 1996 *N* = 1452	Baseline 2006 *N* = 1142	Difference (*p* value)
Age	2594	74.1	74.1	
Female	2594	56.8	56.8	
Co-residing partner (%)	2583	57.0	62.2	0.008
Have children (%)	2288	87.6	89.9	0.103
Income, mean (SD)	2515	2.97 (0.03)	3.07 (0.03)	0.009
Informal care by partner (yes/no; %)	2392	13.0	8.5	<0.001
Informal care by network (yes/no; %)	2392	12.5	12.1	0.813
Formal care (yes/no; %)	2392	9.8	15.2	<0.001
Disability (#), mean (SD)	2557	1.28 (0.05)	1.54 (0.05)	<0.001
Probable dementia (%)	2328	1.1	1.6	0.342
Depressive symptoms, mean (SD)	2536	1.23 (0.05)	1.12 (0.05)	0.100
Lonely, mean (SD)	2382	2.24 (0.07)	2.00 (0.08)	0.027
Chronic lung disease (%)	2590	13.0	13.4	0.777
Heart disease (%)	2589	23.1	28.7	0.002
Peripheral artery disease (%)	2589	9.1	8.8	0.772
Diabetes (%)	2589	7.1	12.4	<0.001
Stroke (%)	2589	5.6	6.9	0.205
Osteoarthritis (%)	2589	44.3	50.6	0.002
Rheumatoid arthritis (%)	2588	9.6	10.3	0.592
Cancer (%)	2589	11.5	15.1	0.009
Other chronic diseases (%)	2591	24.8	26.0	0.505
Incontinence (%)	2591	24.1	28.5	0.020
Hospital visit in last 6 months (yes/no; %)	2387	9.5	11.2	0.187
Two or more chronic diseases	2588	43.4	52.0	<0.001
Three or more chronic diseases	2588	17.8	24.8	<0.001

Percentages and means of 1996 are weighted to 2006 by age and gender

### Explanatory factors of admission rate to institution

Testing the significance of predictors of admission to institutional care per block of factors of the Andersen model, we observe that of the predisposing variables, age and gender were significant predictors at a *p* < 0.10 level; of the enabling variables, this was the case for having a co-residing partner, the neighbourhood income level, informal care by the social network and formal care; of the need variables the disabilities, probable dementia, incontinence, recent hospital visit and diabetes were significant predictors at a level of *p* < 0.10. In the final model, the variables such as gender and income were removed to make the model more parsimonious. These variables were not significant at a *p* = 0.50 level.

The admission rate in the period 2006–2009 was 0.8 % point (CI: 1.0–2.6 %) lower than in the period 1996–1999 (4.5 vs. 5.3 %, or a 15 % decline; see Model I, Table 3). The multivariable regression model shows that the often-reported factors such as age, disability, receiving formal care with household tasks or personal care, a hospital visit in the last 6 months and dementia were significant predictors of admission to an LTC institution (*p* < 0.05; Table 2). Dementia showed the highest odds of admission to an LTC institution. Furthermore, diabetes, having a partner and loneliness were associated with a higher admission rate (*p* < 0.10). The model shows as well a significant “time” effect, which suggests that in the period 2006–2009, less people were admitted to institutional care compared to the period 1995–1999 when they were in a comparable health and personal situation. This effect can be the result of factors such as changes in policy, social values and technology.

**Table 2:** Factors associated with admission to institution, ages 65–89 years (from multivariable logistic regression)

To institution	Model I^a^			Model II		
	Odds ratio	Conf. interval (%)	*p* > |*z*|	Odds ratio	Conf. interval (%)	*p* > |*z*|
Age	1.19	1.07–1.17	<0.001	1.13	1.08–1.17	<0.001
Partner	0.64	0.39–1.06	0.080	0.57	0.34–0.93	0.025
Formal care	2.08	1.25–3.46	0.005			
Informal care by network	1.24	0.71–2.17	0.442			
Hospital visit in last 6 months (yes/no; %)	2.14	1.23–3.70	0.007	2.23	1.29–3.85	0.004
Dementia	36.80	13.47–100.51	<0.001	32.38	11.94–87.81	<0.001
Diabetes	1.74	0.96–3.13	0.066	1.86	1.04–3.33	0.037
Incontinence	1.38	0.87–2.18	0.166	1.45	0.92–2.28	0.110
Disability	1.15	1.02–1.30	0.024	1.20	1.07–1.35	0.002
Lonely	1.07	0.99–1.15	0.082	1.08	1.00–1.16	0.051
Time effect	0.59	0.37–0.96	0.033	0.65	0.41–1.04	0.075
	*N* = 2109, pseudo *R* ^2^ = 0.24			*N* = 2109, pseudo *R* ^2^ = 0.23		

^a^ *Model I* predictors of institutional care after testing blocks of predisposing, enabling and need factors of the Andersen model, *Model II* predictors of institutional care after testing blocks of predisposing, enabling and need factors excluding the potentially endogenous variables formal and informal care

The Blinder–Oaxaca analysis decomposes this difference of 0.8 % in an effect as a result of the difference in the prevalence of poor health and support between the two periods (in Table 3, difference “due to endowments”) and an effect of change in effects of determinants (“due to time effect”). Hence, due to the fact that the sample in 2006–2009 was more disabled and sicker, the probability of admission to an institution of the sample would have *increased* with 1.3 % point (CI −2.1 to −0.6 %) in the period 2006–2009 compared to the period 1996–1999 (see Model I, Table 3). The time effect of 2.1 % point (CI 0.2–4.1 %) indicates that with the same age, health situation and support level, 2.1 % point fewer older adults were admitted to an institution in the period 2006–2009 than in 1996–1999. This amounts to a 40 % decline.

**Table 3:** Decomposition of difference in admission rate to institution between 2006–2009 and 1996–1999

	Model I			Model II		
	%	Conf. interval (%)	*p* > |*z*|	%	Conf. interval (%)	*p* > |*z*|
To institution 1996–1999	5.3	4.1 to 6.4	<0.001	5.3	4.1 to 6.4	<0.001
To institution 2006–2009	4.5	3.2 to 5.8	<0.001	4.5	3.2 to 5.8	<0.001
Difference	0.8	−0.9 to 2.5	0.368	0.8	−1.0 to 2.6	0.371
Due to endowments	−1.3	−2.1 to −0.6	0.001	−0.9	−1.6 to −0.3	0.006
Due to time effect	2.1	0.2 to 4.1	0.031	1.7	−0.2 to 3.6	0.074

*Model I* includes age, partner, formal care, informal care by network, disability, hospital visit in last 6 months, probable dementia, diabetes, loneliness and incontinence, *Model II* includes age, partner, disability, hospital visit in last 6 months, probable dementia, diabetes, loneliness and incontinence

Note that the odds ratios of the variables—formal care and informal care by the social network are above 1. One explanation can be that the caregivers bring the older adults in contact with institutional care (George 1987). A second explanation can be that these variables are endogenous. Firstly, because decisions of older adults and their families concerning formal care, informal care and institutional care are jointly decided. Secondly, because these variables might pick up the effect on admission to institutional care of unobserved differences across individuals and families (Spillman and Long 2009). The golden standard to deal with endogenous variables is to use instrumental variables (IVs). However, we did not find a good candidate for an IV, which might be partly the result of a lack of power. To test the sensitivity of the time effect we ran the logistic regression and Blinder–Oaxaca analysis without the variables—formal care and informal care by the network. The results were largely the same, although the significance level decreased. In the Blinder–Oaxaca analysis, the effect of the endowments is −0.9 % (CI −1.6 to −0.3 %) and the time effect is 1.7 % (CI −0.2 to 3.6 %), a decrease of 32 % (Model II, Table 3).

Furthermore, when we relaxed the selection criterion of the blocks of predisposing, enabling and need variables to be considered for the final model to *p* < 0.20, we observe that the final model is very similar to Model I.

## Discussion

Our results show that the decline in institutional care in the Netherlands in the period 1996–2009 is not the result of changes in need for care. Although the observed difference in the admission rate was relatively small in the period 2006–2009 compared to 1996–1999 (4.5 vs. 5.3 %, a 15 % decrease), the probability of admission in 2006–2009 was relatively much lower as the people at home in 2006 in our sample were overall sicker and more impaired. Our analysis indicates that there is a substantial time effect, suggesting that with the same level of disabilities and chronic diseases and the same support system, the rate of admission to an institution would be 32–40 % lower in 2006–2009 than in 1996–1999 [−2.1 % point (CI 0.2–4.1 %) when the mix of formal and informal care is taken into account to −1.7 % point (CI −0.2 to 3.6 %) when the variables of formal and informal care are not part of the final model]. This time effect might consist of the combined effect of changes in policy (such as more home care or supply factors), technological advances in housing, use of personal alarms and changes in social norms.

Our results support the findings by de Meijer et al. (2015), who conclude that changes in LTC use are not due to shifts in the disability distribution but can almost entirely be traced back to changes in the way the health care system treats disability. Older adults with mild disability are more likely to be treated at home than before, whereas severely disabled individuals continue to receive institutional LTC. Our results are different from research on admission rates of older adults in Germany. After comparing the admission risks of two cohorts of adults over 74 years old, in 1991–1993 and 2002–2003, no time effect was found on nursing home admission in the subsequent 5 years (Braunseis et al. 2012).

The lower admission rate is mirrored by a more disabled and older population in LTC institutions as shown in earlier research (de Klerk 2011). The percentage of people with severe disabilities living in LTC institutions increased from slightly more than 40 % in 2000 to almost 50 % in 2008; the percentage of people that needed a wheelchair increased from 33 % in 2000 to 49 % in 2008; the average age increased from 84 to 85 years and the percentage of adults with a chronic disease increased from 82 % in 2000 to 86 % in 2008.

Strengths of the study are that the data allow us to follow older adults over time and that a broad range of explanatory variables is included. A limitation of the study is the limited number of people admitted to an institution in the follow-up periods and that we do not have data at the time of admission. Hence we cannot know the exact reasons of admission to an institution across the two periods. Furthermore, it cannot be ruled out that the awareness of chronic diseases has changed and has led to a change in the prevalence of chronic diseases. Overreporting of chronic diseases (defined as reported by respondents but not by their GP) became more common in 2008–2009 compared to 1992–1993, whereas underreporting (reported by GP but not by respondent) became less common (Galenkamp et al. 2014). Overall this trend did not result in lower levels of patient–GP agreement on specific chronic diseases in this period. The higher prevalence of chronic diseases over this period seems to be primarily the result of higher survival rates of patients and much less the result of a higher incidence rate (Deeg et al. 2013). If a higher awareness would result in earlier detection of chronic diseases a higher incidence rate can be expected. Except for diabetes these higher incidence rates are not found. In respect of the upward trend in obesity in the Netherlands, the higher incidence rate in diabetes is very plausible: the percentage of adults over 75 years with a body mass index of more than 30 increased from 9.8 % in 1995 to 14.2 % in 2009 (Statistics Netherlands 2015).

This paper shows the difficulty to make any predictions from new policy actions and how these may impact on the admission rates at large. Further research is necessary to disentangle the developments at the macro-level. To be able to make predictions about future need for care, we need to know whether and to what extent the effect of policy, social values and technology play a role and how they reinforce each other. Ideally, future research takes into account changes in these factors over a longer period of time.
